# Bronchial Artery–Pulmonary Artery Fistula With Dual Arterial Feeders Treated Successfully With Staged Embolisation Procedures—A Case Report

**DOI:** 10.1002/rcr2.70401

**Published:** 2025-11-04

**Authors:** Hei‐Shun Cheng, James Fung, K. M. Cyrus Mo, Kevin Chin, Vinson Nelson Yew, Chi‐Chung Jeffrey Wong, Charles Wong, Pui‐Hing Chiu, Chun‐Wai Tong, Pui‐Ling Flora Miu

**Affiliations:** ^1^ Department of Medicine Pamela Youde Nethersole Eastern Hospital Hong Kong Hong Kong; ^2^ Department of Radiology Pamela Youde Nethersole Eastern Hospital Hong Kong Hong Kong

**Keywords:** bronchial artery aneurysm, bronchial artery‐pulmonary artery fistula, endovascular embolisation, left‐to‐right shunt

## Abstract

Bronchial artery‐pulmonary artery fistula (BPAF) is an uncommon vascular malformation with a left‐to‐right shunt. We report a case of a 72‐year‐old woman diagnosed with BPAF with two arterial feeders who had no underlying lung diseases and presented with intermittent haemoptysis. Her digital subtraction angiography confirmed BPAF supplied by the right bronchial artery (BA) from the intercostobronchial trunk and another ectopic accessory right BA originating from the right subclavian artery. Considering the future risk of catastrophic rupture due to high systemic pressure exerted on the fistula, prophylactic embolisation procedures via a percutaneous approach were performed. The pulmonary artery was first embolised for outflow control, followed by coil and microvascular plug embolisation on the right BA. The ectopic accessory right BA was later embolised with coil and microvascular plug as a staged procedure. A 14‐month post‐procedure computed tomography scan confirmed complete thrombosis of both BAs. Our case highlights the efficacy and safety of endovascular embolisation in BPAF.

## Graphical abstract

Bronchial artery‐pulmonary artery fistula (BPAF) with dual arterial feeders is a rare vascular anomaly with the potential risk of catastrophic rupture. Our case report demonstrated the role of endovascular embolisation in preventing massive life‐threatening haemorrhage.

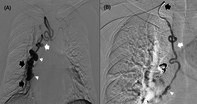

## 1 Introduction

Bronchial artery‐pulmonary artery fistula (BPAF) receives its arterial supply from bronchial artery (BA) with drainage into pulmonary artery. Contrary to the right‐to‐left shunt in pulmonary arteriovenous malformation (PAVM), BPAF demonstrates a left‐to‐right shunt. Given this anatomical distinction, it manifests differently from PAVM both clinically and radiologically. Herein, we report a rare case of BPAF with dual arterial feeders successfully treated with staged embolisation procedures.

## 2 Case Report

A 72‐year‐old Chinese woman, with a history of hypertension, ischaemic heart disease and atrial fibrillation (AF), presented to our hospital with mild intermittent haemoptysis for 3 months. She had no other respiratory or constitutional symptoms. Apart from taking an anticoagulant for embolic stroke prophylaxis, there were no other bleeding tendencies. She had no mucosal bleeding symptoms nor family history of hereditary haemorrhagic telangiectasia (HHT). Physical examination was unremarkable with no signs of telangiectasia. Chest radiograph showed mild cardiomegaly.

**FIGURE 1:**
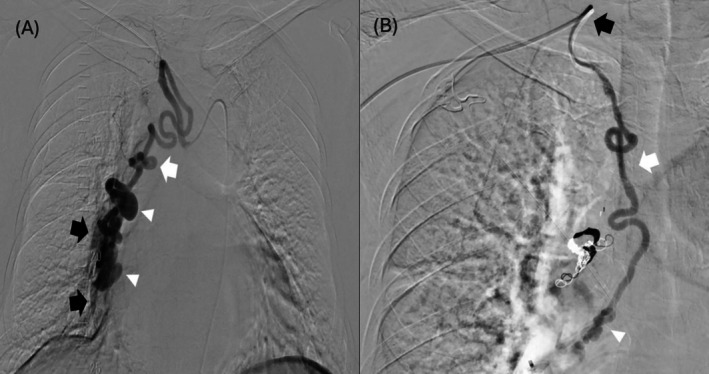
(A) Digital subtraction angiography confirmed a dilated right bronchial artery (BA) (white arrow) arising from the intercostobronchial trunk (ICBT). Multiple aneurysms (white arrowhead) were seen along the dilated and tortuous BA. Early opacification of the RLL pulmonary artery (PA) was observed (black arrow), suggestive of BPAF. (B) An accessory ectopic right BA (white arrow) contributed a minor supply to the BPAF (white arrowhead). It arose from the proximal right subclavian artery (black arrow).

A contrast computed tomography (CT) of the thorax revealed abnormal serpiginous vessels supplied by a dilated and tortuous right BA with multiple aneurysms at the right lower lobe (RLL) of her lung. There was a minor supply from the proximal right subclavian artery. Early opacification of the RLL pulmonary artery was observed. Therefore, a variant form of PAVM was first suspected. An echocardiogram suggested mild pulmonary hypertension with right ventricular systolic pressure (RVSP) of 50 mmHg and preserved left ventricular ejection fraction (56%). There was moderate tricuspid regurgitation but the right heart was not dilated, without right ventricular outflow tract obstruction or intra‐cardiac shunts. Subsequent digital subtraction angiography (DSA) confirmed an abnormal fistula between a dilated right BA and the RLL pulmonary artery (Figure 1A). The dilated BA (4 mm in calibre) received its supply from the intercostobronchial trunk (ICBT) arising from the descending thoracic aorta. Another minor supply to the RLL pulmonary artery was an ectopic accessory right BA (2 mm in calibre) originating from the right subclavian artery (Figure 1B). The diagnosis of BPAF with dual arterial feeders was confirmed.

Our patient's clinical profile was reviewed again for possible secondary causes of BPAF. She was a non‐smoker and had no history of underlying lung diseases such as bronchiectasis, prior tuberculosis infection, chronic airway diseases or congenital lung diseases. She also did not have any remote lung injury or receive any pulmonary surgery in the past. Her lung function test was normal and the CT scan of the thorax did not reveal any lung parenchymal diseases. Her vascular malformation was therefore considered to be a primary BPAF.

**FIGURE 2:**
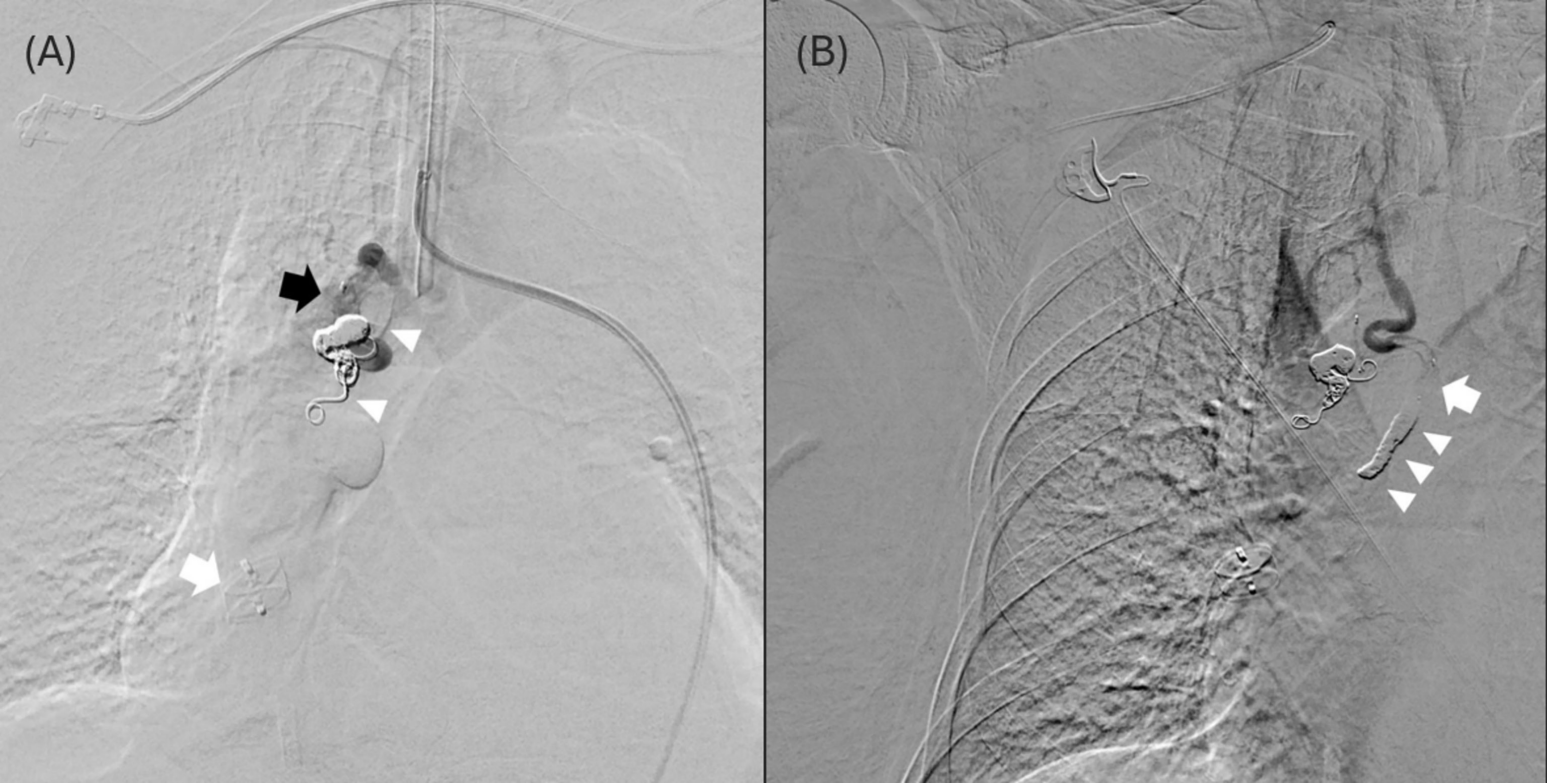
(A) Endovascular embolisation with transfemoral approach. An Amplatzer vascular plug was deployed at the outflow of BPAF at the RLL pulmonary artery (white arrow). Detachable coils (white arrowheads) were then deployed at the right bronchial artery, followed by a microvascular plug (black arrow). (B) Endovascular embolization via ipsilateral radial artery access. Cessation of forward flow to the BPAF was evident after deploying several detachable coils (arrowheads) and a microvascular plug (arrow).

Given the systemic pressure this vascular anomaly subjected to and the presence of BA aneurysms, a definitive treatment was deemed necessary to prevent potentially catastrophic rupture. An elective embolisation of the BPAF under general anaesthesia was arranged. A seven French (Fr.) long vascular sheath (Flexor Raabe guiding sheath, Cook Medical, Indiana, USA) was inserted at the right femoral vein and tracked to the RLL pulmonary artery. The RLL pulmonary artery was embolised with an Amplatzer vascular plug (AVP‐II) (Abbott, Illinois, USA) for outflow control. Another Neuron MAX 088 guiding sheath (Penumbra, California, USA) was tracked to the right BA over a five Fr. Simmons‐1 catheter. The right BA was then embolised with coils (Concerto coils, Medtronic, Minnesota, USA) and a microvascular plug (MVP‐5Q microvascular plug, Medtronic, Minnesota, USA) via a Phenom 27 microcatheter (Medtronic, Minnesota, USA) (Figure 2). Cannulation of the ectopic accessory BA via transfemoral access was difficult. The ectopic accessory right BA was later accessed from the ipsilateral radial artery, using a five Fr. Head‐hunter catheter as a staged procedure. Embolisation was performed with coils (Concerto and Nester coils; Medtronic, Minnesota, USA) and a microvascular plug (MVP‐5Q microvascular plug), via a Rebar‐18 microcatheter and Rebar‐27 microcatheters (Medtronic, Minnesota, USA), respectively.

Our patient remained asymptomatic upon follow‐up. A 14‐month post‐procedure CT showed complete thrombosis of both the right BA and the ectopic accessory right BA.

## 3 Discussion

BPAF is an uncommon thoracic vascular anomaly and a rare cause of bronchial artery hypertrophy [1, 2]. There have been many terminologies describing BPAF, including angioma, hemangioma, fistula or bronchial AVM [1, 2], while bronchial artery racemose hemangioma appears to be a common term in the Japanese literature [3]. Besides BPAF, bronchial artery‐pulmonary vein fistula has also been observed as another variant form of bronchial AVM, resulting in a left‐to‐left shunt [1].

Despite both being high‐flow vascular anomalies, BPAF has several distinctive features that separate it from the well‐described PAVM (Table 1). First, there is no nidus in BPAF. Second, BPAF, as a left‐to‐right shunt, does not share the clinical characteristics of PAVM, which is a right‐to‐left shunt. Patients with BPAF do not have platypnea or orthodeoxia and they are not at risk of embolic stroke or brain abscess. However, they may bear a higher risk of pulmonary hypertension as seen in our patient and catastrophic haemoptysis if rupture occurs. A negative result is also expected for BPAF in shunt fraction evaluation with 100% oxygen prescription and transthoracic echocardiogram with agitated saline contrast.

**TABLE 1:** Comparison of anatomical features, clinical manifestation, complication risk and investigation result between BPAF and PAVM.

	BPAF	PAVM
Feeding blood vessel	Bronchial artery	Pulmonary artery
Draining blood vessel	Pulmonary artery	Pulmonary vein
Source of blood supply	Systemic circulation (Higher pressure)	Pulmonary circulation (Lower pressure)
Shunt direction	Left‐to‐right shunt	Right‐to‐left shunt
Platypnea/orthodeoxia	Absent	Present
Haemoptysis	Potentially catastrophic if BPAF ruptures	Less severe than BPAF if PAVM ruptures
Association with hereditary haemorrhagic telangiectasia	No	Yes
Risk of brain abscess or embolic stroke	No	Yes
Risk of pulmonary hypertension	Higher	Lower
Shunt fraction evaluation with 100% oxygen	Negative test	Positive test
Transthoracic echocardiogram with agitated saline contrast	Negative test	Positive test

Abbreviations: BPAF, bronchial artery‐pulmonary artery fistula; PAVM: pulmonary arteriovenous malformation.

The aetiology of BPAF could be primary (i.e., congenital) or secondary to underlying lung disease such as chronic airway diseases, pulmonary infection, malignancy and interstitial lung diseases. History of lung transplantation or traumatic lung injury could serve as a remote cause of BPAF [1, 2, 4]. A detailed history taking and physical examination are necessary to screen for any secondary causes, followed by imaging studies such as CT scan of the thorax. Lung function tests are sometimes required to look for obstructive airway or restrictive lung diseases. Patients could present with haemoptysis or dyspnoea. The dilated BA in BPAF is sometimes accompanied by BA aneurysms. Together they may compress the left recurrent laryngeal nerve, causing hoarseness of voice [5], simulate submucosal lesions of the oesophagus [6] or even manifest as non‐pulsatile nodules in the bronchial tree [1], of which biopsy by the unwary may cause life‐threatening haemorrhage [1].

Radiologically, BPAF appears as serpiginous vessels supplied by a dilated BA commonly found in the right middle or lower lobe [2] with multifaceted manifestation. It could simulate a hilar mass on a chest radiograph with the presence of a large BA aneurysm [7] or even pulmonary embolism on a CT pulmonary angiogram due to washout by unopacified blood from the BA during the pulmonary phase [4].

There is no international consensus on BPAF treatment. Prophylactic embolisation is usually recommended to prevent life‐threatening haemorrhage as the size of the BA aneurysm is not correlated with rupture risk and BPAF is subjected to highly pressurised systemic circulation [8]. The decision for embolisation in this case was based on the patient's clinical profile and symptoms. Various embolic agents, including coils, vascular plug and liquid embolics such as N‐butyl cyanoacrylate (NBCA) glue are used for treating BPAF. More advanced techniques have been introduced including proximal flow control using microballoon [9] and access via percutaneous approach. In cases where BA aneurysms arise closely from the thoracic aorta, coil embolisation with endovascular stent graft deployment can be considered [8]. Given the rarity of BPAF, no single embolic agent has demonstrated superiority over the others. The course of BPAF is usually highly tortuous. There were several aneurysmal dilatations in our case, suggesting potential vulnerable points in the lesion. Although the authors attempted to embolise the BPAF as close as possible to the site of the fistula in order to minimise the risk of recurrence, the anatomy of the BPAF in this case did not allow safe and stable distal cannulation. No imaging or clinical evidence of pulmonary infarct was noted on the follow‐up scan. Overall, despite a limited body of robust evidence, image‐guided embolisation is a safe and minimally invasive treatment option compared to surgical ligation or pneumonectomy, which carries a higher morbidity and mortality rate.

The BPAF in our case was likely primary as our patient had no known lung diseases and her lungs were unremarkable on CT. Given the high‐flow left‐to‐right shunt, we first embolised the pulmonary artery to control the outflow of this vascular anomaly, thereby minimising the risk of non‐target embolisation. This mirrors the approach described by Pua U et al. [3]. For the BA embolisation, we used both detachable coils and a microvascular plug. This method of coiling followed by plugging is thought to provide a more secure and permanent occlusion by a few authors [10].

In conclusion, our case highlights the distinct clinical and radiological features of BPAF and the role of endovascular embolisation in preventing massive life‐threatening haemorrhage.

## Author Contributions

Hei‐Shun Cheng, James Fung, K. M. Cyrus Mo and Kevin Chin contributed to conceptualization, data collection, data interpretation, drafting and editing of the manuscript. Vinson Nelson Yew and Chi‐Chung Jeffrey Wong contributed to conceptualization and drafting of the manuscript. Charles Wong, Pui‐Hing Chiu, Chun‐Wai Tong and Pui‐Ling Flora Miu contributed to conceptualization and editing of the manuscript. All authors validated the final version of the manuscript.

## Consent

The authors declare that written informed consent was obtained for the publication of this manuscript and accompanying images using the consent form provided by the Journal.

## Conflicts of Interest

The authors declare no conflicts of interest.

## Data Availability

The data that support the findings of this study are available from the corresponding author upon reasonable request.
